# Short range magnetic correlations in van der Waals 2D materials analyzed using neutron scattering

**DOI:** 10.1063/4.0001015

**Published:** 2025-10-27

**Authors:** Raju Baral, Amanda A. Haglund, Jue Liu, Alexander I. Kolesnikov, David Mandrus, Stuart Calder

**Affiliations:** 1 Oak Ridge National Laboratory, Oak Ridge, TN, USA; 2 University of Tennesse, Knoxville, TN, USA

## Abstract

Two-dimensional layered materials, where magnetic layers are linked through van der Waals (vdW) bonding provides a promising platform for spintronics applications and quantum behavior. However, realizing their full potential requires a deeper understanding of their spin behavior across different length scales. In this study, we investigated the local magnetic correlations of bulk antiferromagnetic vdW materials MnPSe_3_, MnPS_3_ and CrPS_4_ using neutron scattering using the magnetic pair distribution function (mPDF) technique. We explore short-range magnetic correlations in a systematic series of MnPS_x_Se3_-x_ (x=0, 1, 1.5, 2,3) powder samples with neutron total scattering data. Our results reveal that substituting S/Se anions, despite being non-magnetic, tunes both atomic structure and enables the gradual modulation of magnetic correlation length and spin angle. Complementary inelastic neutron scattering measurement further quantify changes in the magnetic exchange interactions, highlighting the continuous evolution of spin correlations across the series. Additionally, short-range magnetic correlations of CrPS_4_ were analyzed and modeled using the mPDF technique in the paramagnetic regime, under both zero-field and applied magnetic field conditions.

